# The Frontal Eye Field Is Involved in Visual Vector Inversion in Humans – A Theta Burst Stimulation Study

**DOI:** 10.1371/journal.pone.0083297

**Published:** 2013-12-20

**Authors:** Katrin Jaun-Frutiger, Dario Cazzoli, René M. Müri, Claudio L. Bassetti, Thomas Nyffeler

**Affiliations:** 1 Perception and Eye Movement Laboratory, Departments of Neurology and Clinical Research, Inselspital, Bern University Hospital, and University of Bern, Bern, Switzerland; 2 Nuffield Department of Clinical Neurosciences, University of Oxford, John Radcliffe Hospital, Oxford, United Kingdom; 3 Centre of Neurology and Neurorehabilitation, Luzerner Kantonsspital, Luzern, Switzerland; University of British Columbia, Canada

## Abstract

In the antisaccade task, subjects are requested to suppress a reflexive saccade towards a visual target and to perform a saccade towards the opposite side. In addition, in order to reproduce an accurate saccadic amplitude, the visual saccade vector (i.e., the distance between a central fixation point and the peripheral target) must be exactly inverted from one visual hemifield to the other. Results from recent studies using a correlational approach (i.e., fMRI, MEG) suggest that not only the posterior parietal cortex (PPC) but also the frontal eye field (FEF) might play an important role in such a visual vector inversion process. In order to assess whether the FEF contributes to visual vector inversion, we applied an interference approach with continuous theta burst stimulation (cTBS) during a memory-guided antisaccade task. In 10 healthy subjects, one train of cTBS was applied over the right FEF prior to a memory-guided antisaccade task. In comparison to the performance without stimulation or with sham stimulation, cTBS over the right FEF induced a hypometric gain for rightward but not leftward antisaccades. These results obtained with an interference approach confirm that the FEF is also involved in the process of visual vector inversion.

## Introduction

In the antisaccade task, subjects are requested to suppress a reflexive saccade towards a visual target and to shift their gaze towards the opposite side. In order to accurately produce antisaccades with the same amplitude, the vector leading to the presented target location must be precisely inverted by 180° into an opposite saccade vector. The posterior parietal cortex (PPC) has consistently been shown to be involved in this visual vector inversion process. In the monkey, a subset of neurons in the lateral intraparietal area (LIP) shows an early activity when the visual stimulus matches the contralateral receptive field of the cell and, later on, shows a “paradoxical” activity when the visual target is presented ipsilaterally [1]–[2]. In the human, evidence for the fact that the PPC is involved in visual vector inversion arises from several studies applying correlational approaches such as event-related potentials [3], functional magnetic resonance imaging (fMRI) [4], and magnetoencephalography (MEG) [5]. Moreover, it has been shown that interference with the activity of the PPC with transcranial magnetic stimulation (TMS) [6] or a focal lesion of the PPC [7] directly disturbs visual vector inversion, triggering a marked hypometria of ipsilateral antisaccades.

Another oculomotor cortical area that is involved in the processing of visual information and saccade generation – and might thus be implied in visual vector inversion – is the FEF [8]–[10]. In fact, previous studies applying correlational approaches (i.e., fMRI, MEG) have shown similar activities in the FEF and in the PPC during visual vector inversion [5], [11]–[12]. However, to date, there are no human FEF lesion studies that have analyzed the involvement of this oculomotor cortical area in visual vector inversion per se. A method that allows to circumvent the issue of the lack of lesion studies is represented by the application of an “offline” interference approach such as continuous theta burst stimulation (cTBS). cTBS is a repetitive transcranial magnetic stimulation (TMS) protocol that has been shown to induce inhibitory behavioural effects lasting up to 30 minutes [13]. The advantage of such an approach is that a temporary “functional lesion” of the FEF can be induced, and the process of visual vector inversion can be assessed in an antisaccade task offline (i.e, several minutes after stimulation application). In the present study, we aimed at clarifying whether the FEF is involved in visual vector inversion applying cTBS. If the FEF is similarly involved in visual vector inversion as the PPC, then cTBS over the right FEF should trigger the same deficits in antisaccades as observed after a lesion of the right PPC [7], namely hypometric rightward antisaccades. To assess this hypothesis, ten subjects were tested with a task in which they had to perform antisaccades as accurately as possible. Saccadic gain was measured under three different conditions: without any stimulation, after cTBS over the right FEF, and after sham stimulation over the right FEF.

## Methods

### Ethics statement

The study was approved by the Ethical Committee of the State of Bern and was carried out in accordance with the principles of the latest Declaration of Helsinki.

### Subjects

Ten right-handed subjects volunteered for the study (six females and four males). Their mean age was 29 years (range 24–33 years). All participants had normal or corrected-to-normal visual acuity and gave their written informed consent prior to participation.

### Eye movement recording and saccade paradigm

Subjects were seated in a completely dark room, their head was stabilised by a chin rest. The chin rest minimised head movements and ensured a constant viewing distance of 120 cm. Visual stimuli were presented on a vertical panel with an embedded array of separate and equidistant light emitting diodes (LED), positioned along the horizontal meridian. Eye movements were recorded by means of an infrared corneal reflection device (Iris Skalar, Delft, Netherlands), with a spatial resolution of 0.1° and sampling rate of 1000 Hz. Eye movement data were stored on a computer for off-line analysis. The device was calibrated at the beginning and regularly throughout the experiment. During calibration, subjects were instructed to look at lateral targets appearing in a staircase pattern, first to the right and then to the left, with amplitudes of 8°, 10°, 12°, 14°, and 16°.

At the beginning of each trial of the memory-guided antisaccade task [6], a central fixation point was presented. After a pseudo-randomized duration of 1500 to 2900 ms, a lateral target was presented for 250 ms on the left or on the right, with pseudo-randomized amplitudes (8°, 10°, 12°, 14°, or 16° from the central fixation point). After a delay of 1000 ms, the central fixation point extinguished. This was the “go” signal for the subjects to perform a saccade towards the mirrored location of the peripheral target. Subjects were instructed to perform the antisaccade task as accurately as possible, not as fast as possible (i.e., the importance of accuracy and not of speed was stressed) after disappearance of the central fixation point. After further 1000 ms, a mirror-positioned target (i.e., a target positioned at the exact opposite location of the previous lateral target) was presented, in order to allow for a corrective saccade, where necessary.

### Stimulation procedure

cTBS was applied using a MagPro R30 stimulator (Medtronic Functional Diagnostics, Skovlunde, Denmark), connected to a figure-of-eight coil (Magnetic coil Transducer MC-B70, Medtronic Functional Diagnostics). For the sham condition, a sham coil (Magnetic Coil Transducer MC-P-B70; Medtronic Functional Diagnostics) was used. The stimulator was set to produce repetitive, biphasic pulses. Stimulation intensity was then set at 80% of the participants' individual resting motor threshold of the left small hand muscles. This stimulation intensity has been shown to be sufficient to induce behavioural effects when applied over the FEF [14]–[15]. The adapted cTBS protocol was the same as described previously [13], [16]–[19]. One continuous train of cTBS with 801 pulses was delivered in 267 bursts, each burst consisting of three pulses at 30 Hz, repeated at intervals of 100 ms. The total duration of one single cTBS train was 44 s. The right FEF was localized as previously described [20]–[21]. In brief, the individual resting motor threshold of the left small hand muscles was determined in every subject. The handle was then moved anteriorly with respect to the hand area, 2–3 cm on average. The handle of the coil was pointed backwards with an angle of 45° with respect to the participants' sagittal plane. The subjects performed the whole experiment immediately after the application of one single continuous cTBS train.

### Experimental procedures

For each condition (i.e., without stimulation, cTBS FEF, sham TBS FEF), the subjects performed 100 trials overall, 50 towards the left (i.e., lateral target on the right) and 50 towards the right (i.e., lateral target on the left). The order of the conditions was pseudo-randomized across subjects. The experiment lasted about 20 min for each condition. The different conditions were performed during three different sessions, with an interval of at least 24 hours between sessions.

### Data analysis

In a first step, erroneous prosaccades (i.e., saccades executed towards the lateral target rather than towards its mirrored position), anticipated saccades (i.e., saccades executed before the “go” signal represented by the disappearance of the central fixation point and/or saccades that started outside an area of 1 degree around the central fixation point), and trials with blinks were excluded from the main analysis. The percentage of erroneous prosaccades, anticipated saccades, and trials with blinks was computed for every subject and condition. The values were then compared between conditions by means of separate, repeated-measures analyses of variance (ANOVA) with the within-factor ‘condition’ (levels: cTBS right FEF; sham right FEF; no stimulation).

For each correctly executed antisaccade, gain 1 was calculated by dividing the amplitude (in degrees) of the executed antisaccade by the amplitude (in degrees) of the lateral target. Gain 2 – defined as the gain of the saccade before the mirror-positioned feedback target was presented – was calculated according to the same formula. Mean gain 1 and mean gain 2 were computed for each subject, stimulation condition, and direction. The values were then analysed by means of separate, repeated-measures ANOVA with the within-factors ‘condition’ (levels: cTBS right FEF; sham right FEF; no stimulation) and ‘direction’ (levels: rightward; leftward).

All *post-hoc* tests were computed by means of Bonferroni-corrected t-tests.

## Results

Erroneous prosaccades occurred in 2.768% (standard error of the mean (SEM) = .930) of cases in the condition ‘no stimulation’, in 3.537% (SEM = 1.857) of cases in the condition ‘sham right FEF’, and in 5.001% (SEM = 1.817) of cases in the condition ‘cTBS right FEF’. There was no significant difference between conditions (F_2,18_ = 2.303, *p* = .129, Partial Eta squared (η^2^ *_p_*) = .204). Anticipated saccades occurred in 7.377% (SEM = 2.979) of cases in the condition ‘no stimulation’, in 8.478% (SEM = 3.438) of cases in the condition ‘sham right FEF’, and in 8.938% (SEM = 3.700) of cases in the condition ‘cTBS right FEF’. There was no significant difference between conditions (F_2,18_ = .861, *p* = .439, η^2^ *_p_* = .087). Trials with blinks occurred in 4.250% of cases (SEM = 1.655) in the condition ‘no stimulation’, in 5.320% of cases (SEM = 1.656) in the condition ‘sham right FEF’, and in 4.793% (SEM = 1.147) of cases in the condition ‘cTBS right FEF’. There was no significant difference between conditions (F_2,18_ = .223, *p* = .803, η^2^ *_p_* = .024).

**Figure 1:**
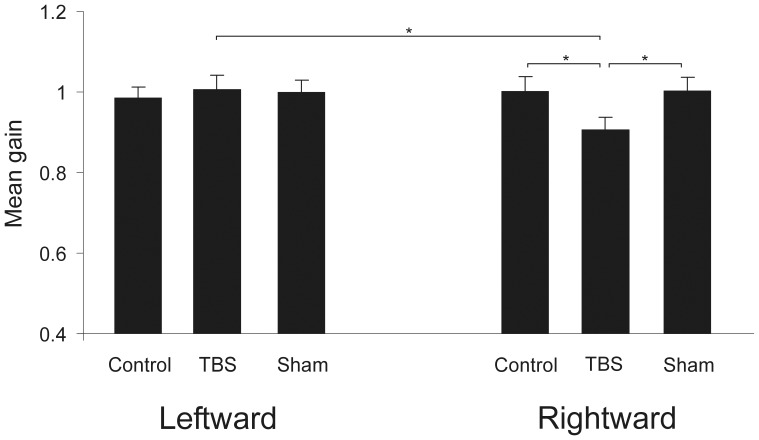
Mean gain 1 (error bars: +/− 1 standard error of the mean (SEM)) of antisaccades for the three stimulation conditions (cTBS right FEF; sham right FEF, no stimulation) and the two directions (leftward; rightward). cTBS over the right FEF induced a significantly hypometric gain for rightward antisaccades, but not for leftward ones (* indicate significant post hoc tests, Bonferroni-corrected; rightward control vs. rightward TBS: *p* = .011; rightward sham vs. rightward TBS: *p* = .003; leftward TBS vs. rightward TBS: *p* = .012).

The analysis of mean gain 1 revealed no significant main effect of the factor ‘condition’ (F_2,18_ = 2.465, *p* = .113, η^2^ *_p_* = .215) or of the factor ‘direction’ (F_1,9_ = .092, *p* = .768, η^2^ *_p_* = .010). That is, the stimulation condition or the direction of the antisaccades *per se* had no significant influence on mean gain 1. However, there was a highly significant interaction between the two factors (‘condition x direction’: F_2,18_ = 10.115, *p*<.001, η^2^ *_p_* = .529). *Post-hoc* testing revealed that mean gain 1 for rightward memory-guided antisaccades (i.e., when the lateral target was presented on the left side) was significantly lower in the condition ‘cTBS right FEF’ (m = .914, SEM = .032) than in the condition ‘no stimulation’ (m = 1.019, SEM = .036; *p* = .011) and the condition ‘sham right FEF’ (m = 1.032, SEM = .032; *p* = .003). Conversely, there was no significant difference between mean gain 1 of leftward antisaccades (i.e., when the lateral target was presented on the right side) in the conditions ‘no stimulation’ (m = .973, SEM = .031), ‘cTBS right FEF’ (m = 1.018, SEM = .034), or ‘sham right FEF’ (m = 1.004, SEM = .033) (all *p*'s>.05). Moreover, there was a significant difference between mean gain 1 of leftward and rightward antisaccades in the condition ‘cTBS right FEF’ (*p* = .012), but not in the conditions ‘no stimulation’ or ‘sham right FEF’ (*p*'s>.05). The results are depicted in Figure 1.

The analysis of mean gain 2 revealed similar results. There was no significant main effect of the factor ‘condition’ (F_2,18_ = 1.490, *p* = .252, η^2^ *_p_* = .142) or of the factor ‘direction’ (F_1,9_ = 1.874, *p* = .204, η^2^ *_p_* = .172). However, there was a significant interaction between the two factors (‘condition x direction’: F_2,18_ = 5.893, *p* = .011, η^2^ *_p_* = .396). *Post-hoc* testing revealed a significant difference between mean gain 2 of leftward and rightward antisaccades in the condition ‘cTBS right FEF’ (*p* = .014), but not in the conditions ‘no stimulation’ or ‘sham right FEF’ (*p*'s>.05). However, mean gain 2 for rightward memory-guided antisaccades (i.e., when the lateral target was presented on the left side) was not significantly different between the conditions ‘cTBS right FEF’ (m = .962, SEM = .021), ‘no stimulation’ (m = 1.044, SEM = .032), or ‘sham right FEF’ (m = 1.028, SEM = .024) (all *p*'s>.05). There was also no significant difference in mean gain 2 of leftward antisaccades (i.e., when the lateral target was presented on the right side) between the conditions ‘no stimulation’ (m = 1.028, SEM = .030), ‘cTBS right FEF’ (m = 1.063, SEM = .022), or ‘sham right FEF’ (m = 1.035, SEM = .033) (all *p*'s>.05).

Hence, cTBS applied over the right FEF specifically triggered significantly hypometric rightward antisaccades.

## Discussion

The results of our study show that cTBS applied over the right FEF induces a significant hypometria of rightward memory-guided antisaccades, whereas the metrics of leftward memory-guided antisaccades are not affected. An interference with the function of the right FEF thus provoked a similar pattern of modification of the antisaccade gain as the one observed after a focal lesion over the right PPC [7].

The finding that a “virtual lesion” of the FEF can induce an ipsilateral effect – characterized by hypometric antisaccades – is particularly interesting, since the FEF is traditionally seen as controlling the motor aspects of contralateral saccades [22].

Our results thus corroborate the findings of recent studies applying correlational approaches and suggesting that the FEF might be implied in visual vector inversion (i.e., in inverting the visual vector between the fixation point and the peripheral target from the contralateral to the ipsilateral visual hemifield). For instance, in a MEG study [5], the FEF showed a similar pattern of activity as the PPC during the execution of antisaccades. Other studies using fMRI showed a higher activity during antisaccade tasks than during prosaccade tasks, not only in the PPC, but also in the FEF [11]–[12]. The possible involvement of the FEF in visual vector inversion in antisaccades has also been postulated in animal studies. In the monkey FEF, neurons have been described, which show a movement of their receptive field that anticipates the visual consequences of planned saccades [23]. Furthermore, using a singleton search task with prosaccades and antisaccades, Sato and Schall [9] found that visual selection and saccade selection are two distinguishable processes of the FEF. In a subsequent study with a prosaccade/antisaccade paradigm, Schall [10] examined the activity of FEF neurons when the singleton fell in the neuron's receptive field and when the singleton was located opposite the receptive field. He could show that on antisaccades trials some neurons initially selected the singleton, but subsequently a transition occurred whereby the endpoint of the antisaccades was selected.

It remains unclear whether visual vector inversion relies more prominently on the FEF or the PPC, and it is not known how the processes of visual perception and spatial working memory are exactly linked to visual vector inversion. It is well known that a subpopulation of FEF neurons encodes visual stimuli in a retinotopic coordinate system [24]–[26], even when the monkey shifts its gaze away as in the antisaccade paradigm [10]. It might thus be possible that in our study cTBS deranged this coordinate system, resulting in the encoding of a shortened vector of the left stimulus. Monkeys have been shown to have difficulties in acquiring targets beyond 15° of eccentricity from straight ahead after a reversible inactivation of the FEF [27]. Additionally, the FEF is involved in spatial working memory [28]. Since in our paradigm we used a delay of 1000 ms before the “go” signal for the execution of the antisaccade was given, it might theoretically be possible that cTBS could have interfered with this memory process. From a recent single-pulse TMS study [6], we know that visual vector inversion during a memory-guided antisaccade paradigm occurs very early in the PPC, i.e., 100 ms after target onset. The vector inversion signal is then transferred transcallosally to the ipsilateral oculomotor network for memorizing and motor planning of the antisaccade. If we now speculate that the vector of a visual stimulus is perceived and inverted in a contralateral network involving both the PPC *and* the FEF, it might be conceivable that visual vector inversion occurs analogously in the PPC and the FEF, i.e., very early. Future studies should shed more light on the temporal aspects of the FEF involvement in visual vector inversion, and compare them with the PPC, e.g., using a correlational approach (fMRI, MEG) or a single-pulse TMS interference approach.

Summing up, the present study confirms that also the FEF, and not only the PPC, is an important oculomotor cortical area for visual vector inversion in humans. Since the FEF and the PPC are densely interconnected [29]–[30], it might be conceivable that a parieto-frontal network might be implied in the control of visual vector inversion. In line with this assumption, both the FEF and the PPC are well know to be involved in mental rotation [31]–[32], a cognitive process that requires a rotational transformation and thus incorporates visual vector inversion.
